# Mixed-mode Zentangle and Pastel Nagomi artwork for improving mental well-being in university students during COVID-19 pandemic – a randomized controlled feasibility trial

**DOI:** 10.3389/fpsyg.2023.1132923

**Published:** 2023-05-24

**Authors:** Kin Cheung, Ka Yan Ma, Hilda Tsang, Nok Hang Leung, Ka Yee Lui, Sze Wing Ho

**Affiliations:** The Hong Kong Polytechnic University, Kowloon, Hong Kong SAR, China

**Keywords:** Pastel Nagomi art, Zentangle art, art-based intervention, mental health, undergraduates

## Abstract

**Background:**

University students are identified as a high-risk group for mental health problems. Artworks have been found effective in enhancing individuals’ mental well-being in different populations, but none have been conducted on university students. This study was to address this research gap to determine the feasibility and estimate the preliminary effects of Zentangle and Pastel Nagomi on the mental well-being of undergraduate students during the COVID-19 pandemic.

**Method:**

This was a 3-arm randomized controlled trial, with 33 undergraduates allocated to two 8-week artworks (Zentangle or Pastel Nagomi Art group) and a control group. Data were collected at baseline, and weeks 4, 6, 8, and 12. Focus group interviews were conducted at the 12-week follow-up.

**Results:**

The consent and attrition rates were 80.5 and 6.06%, respectively. The attendance rate ranged from 83.3 to 100%. Compared with the control group, the Pastel Nagomi art group had a significant improvement in retaining positive affect at week 6. This retention could be further observed at week 12. Moreover, the Zentangle group had a significant increase in positive affect at week 4, with better retention at week 12. In addition, the within-group analyses showed that the Pastel Nagomi art group had significantly decreased negative affect at weeks 6 and week 12; and the Zentangle group had significantly decreased depression at week 8. The qualitative findings suggested that the intervention resulted in the participants enjoying the artwork process, and being proud of their artwork and personal growth.

**Limitation:**

The study included an imbalance number of online vs. face-to-face sessions, and repeated measures may have affected the results.

**Conclusion:**

The study suggests that both artworks are effective in improving undergraduates’ mental well-being and that it is feasible to conduct future large-scale studies (263 words).

## 1. Introduction

Worldwide, the COVID-19 pandemic has created a crisis for mental health. The World Health Organization (WHO) has called for mental well-being to be a global priority (World Health Organization, 2020). University students have been identified as a high-risk group, suffering from psychological symptoms even before the pandemic (Wang, 2009; Limone and Toto, 2022). The social distancing policy has escalated learning with technology, creating a “new normal” for education. However, the proliferation of online teaching and learning and the pandemic-associated restrictions have led to further deteriorations in students’ mental health, because of decreasing social interaction, delayed academic activities, and reduced opportunities to seek counselling services (Cao et al., 2020; Li et al., 2020; Ando, 2021; Jojoa et al., 2021; Chen and Lucock, 2022). An artwork intervention program could be one of the alternatives to improve university students’ psychological states.

### 1.1. Artwork interventions

In recent years, expressive arts interventions, such as artwork, music, drama, narrative and storytelling, have been found to be effective in enhancing individuals’ psychological well-being (Phillips and Becker, 2019). According to a recent systematic review of expressive art interventions for health workers (Phillips and Becker, 2019), artwork and music interventions have had greater positive impacts on mental health than storytelling or narrative. Moreover, artwork intervention can work as a mindfulness-based therapy model to bring positive effects such as stress reduction, emotional regulation, self-esteem, self-awareness and resilience (Coholic, 2011). Specifically, the evidence about face-to-face artwork interventions has shown improved psychological well-being in different settings, including counsellors in the United States (Ifrach and Miller, 2016), nurses and nursing assistants in Lithuania (Karpavičiūtė and Macijauskienė, 2016; Hsu et al., 2021) and end-of-life workers in Hong Kong (Potash et al., 2014).

Among undergraduates, a few studies have examined the relationship between face-to-face art-based workshops and psychological state (Curry and Kasser, 2005; Walsh et al., 2005; Sandmire et al., 2012; Campenni and Hartman, 2020; Sonnone and Rochford, 2020). Anxiety could be reduced by creative art (Walsh et al., 2005), free-form painting (Sandmire et al., 2012), or coloring mandalas (Curry and Kasser, 2005; Walsh et al., 2005; Sandmire et al., 2012; Campenni and Hartman, 2020). Walsh et al. (2005) found that creative art reduced stress and increased positive emotion. Sonnone and Rochford (2020) found that group art therapy could improve undergraduates’ ability to express and disclose themselves, enhance their social connections, and help them to develop new insights. The benefits of artwork in improving psychological status among undergraduates via face-to-face modes have been found. However, considering the “new normal’ of online teaching during the COVID-19 pandemic and a post-pandemic future, the effectiveness of online artwork workshops on improving undergraduates’ psychological well-being worth exploring.

### 1.2. Zentangle and Pastel Nagomi artworks

The popularity of the Zentangle method and Pastel Nagomi Art is growing. These two art forms share similar concepts. For example, they promote relaxation by emphasizing no comparisons, critiques, or judgments of the finished piece with others, and appreciating the opportunity to make choices [Japan Pastel Hope Art Association (JPHAA), 2003; Krahula, 2012]. Furthermore, both only require simple materials and little space.

#### 1.2.1. Zentangle

**Figure 1:**
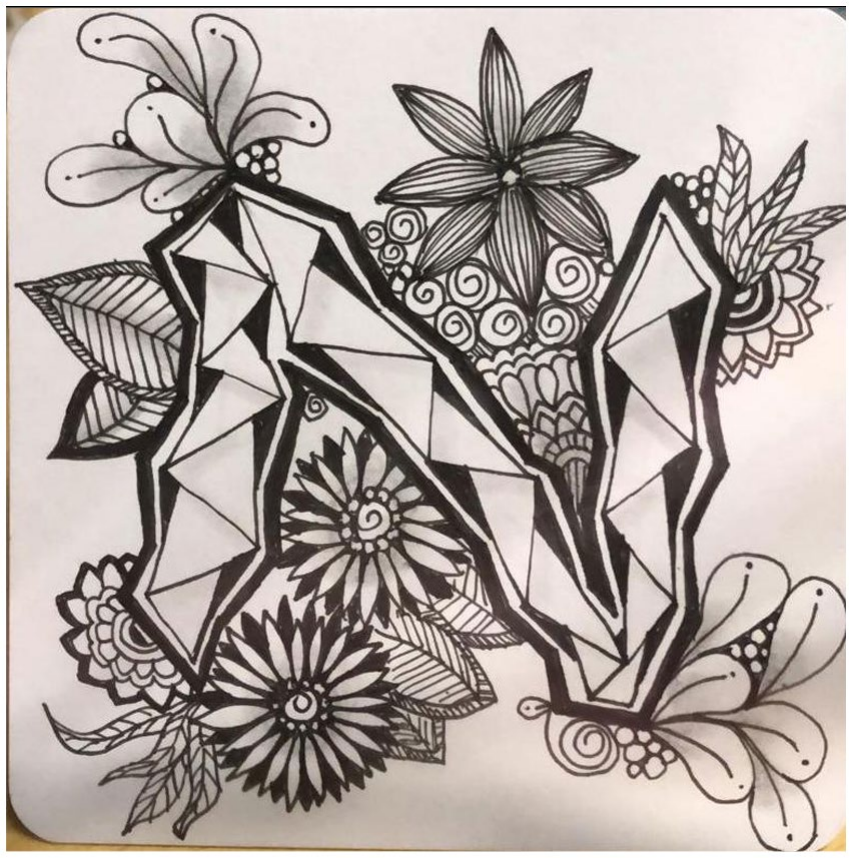
An example of Zentangle Art work drawn by the participant.

Zentangle was developed by Rick Roberts and Maria Thomas (Krahula, 2012) in the United States. According to the official website of Zentangle, the technique was founded in 2003, with significant growth over a short period of time. There are currently over 3,000 Certified Zentangle Teachers (CZT) in 40 countries (Zentangle Inc, n.d.). It is easy to learn and aligns with mindfulness-based stress reduction interventions (Moore, 2013). Relaxation effects can be created by drawing structured patterns repeatedly; these patterns consist of dots, lines, simple curves, S-curves and orbs (Krahula, 2012). This method has been reported as having a positive association with improved mental well-being in psychosis patients in Taiwan (Chen et al., 2016), primary school students in Taiwan (Chia et al., 2020), healthcare workers in Taiwan (Hsu et al., 2021), and first-year university teacher students in Malaysia (Hui and Marof, 2019). Chen et al. (2016) reported that Zentangle significantly improved psychosis patients’ (*n* = 22 in intervention and *n* = 22 in control groups) social interaction anxieties and self-esteem through eight weekly one-hour workshops. Chia et al. (2020) associated the Zentangle method with reducing stress and calming the minds of primary school students (*N* = 12) through eight weekly 75-min drawing workshops. Hsu et al. (2021) found that it helped to improve healthcare workers’ psychological well-being by reducing stress, workplace stress and frustration, enhancing self-efficacy and increasing their commitment to work (*N* = 40) from just one four-hour workshop. Hui and Marof (2019) reported that the Zentangle method increased the positive affect of the university students (*N* = 44) with four three-hour workshops. A review of the literature found four mentioned relevant studies, mainly with pre-and post-single group study designs (Chen et al., 2016; Hui and Marof, 2019; Chia et al., 2020; Hsu et al., 2021). Although studies of the Zentangle’s effectiveness are limited, the results are promising. More studies conducted in different countries with randomised controlled trials and adequate sample size are necessary to validate its effectiveness. An example of Zentangle art is shown in Figure 1.

#### 1.2.2. Pastel Nagomi art

**Figure 2:**
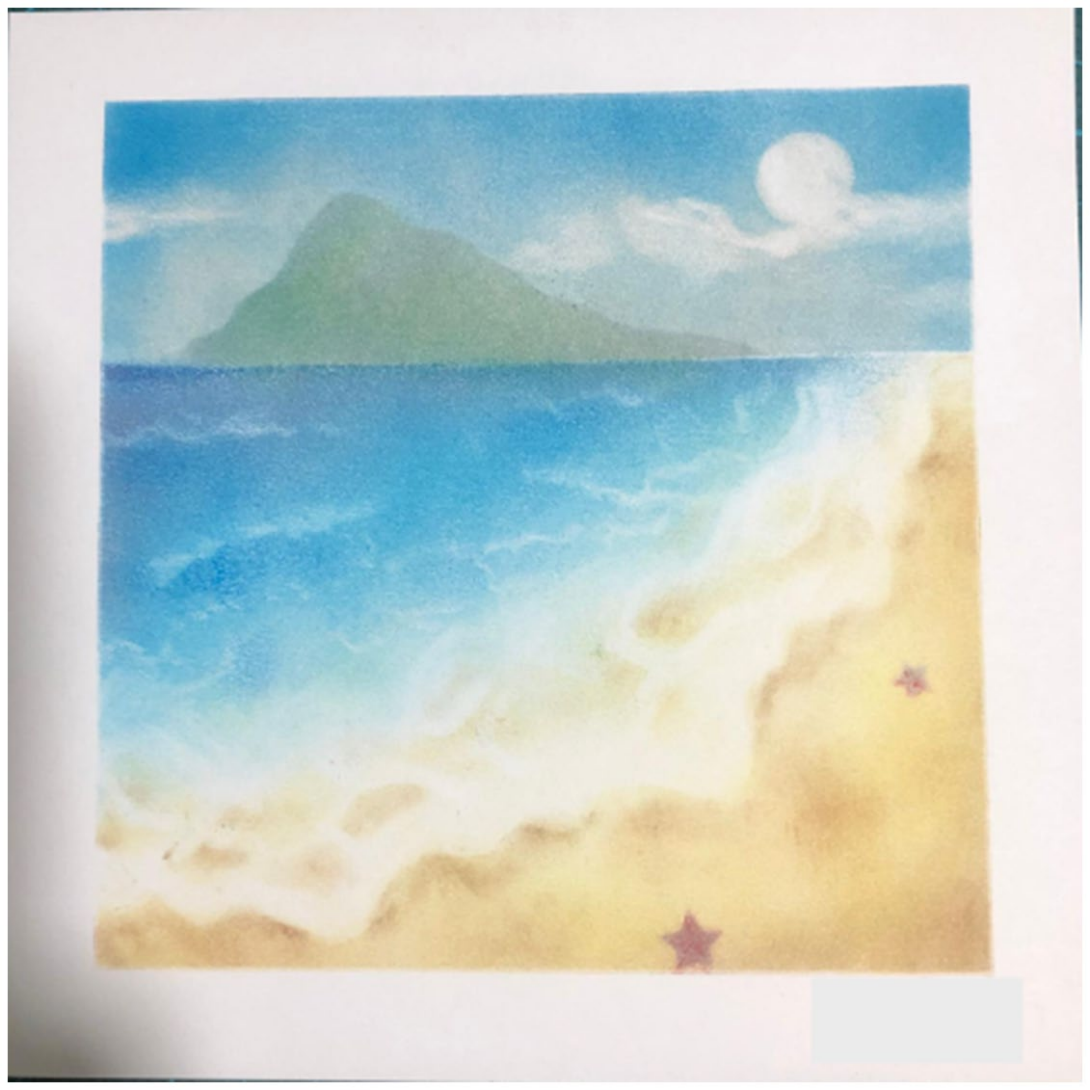
An example of Pastel Nagomi Art work drawn by the participant.

Pastel Nagomi Art was developed by Hosaya Norikatsu in Japan Pastel Hope Art Association (JPHAA) (2003) in Japan. It is also described as “Hope Art.” It involves using fingers to apply powered pastels directly onto the paper. It requires no specific training or talent. Similar to the Zentangle method, Pastel Nagomi Art imposes no restrictions or rules of “correctness,” which encourages individuals to self-reflect and increase their self-acceptance, hence helping to enhance self-esteem and self-efficacy [Japan Pastel Hope Art Association (JPHAA), 2003]. Because of its simplicity and colourful drawings, Pastel Nagomi Art has become popular in Asia, particularly in Hong Kong where numerous tertiary institutions and non-profit organizations arrange Pastel Nagomi Art workshops to enhance the mental health and well-being of the general public. To our knowledge, there have not been any studies examining the effectiveness of the Pastel Nagomi Art on mental health. An example of Pastel Nagomi Art is shown in Figure 2.

To fill the research gaps, this research aimed to conduct a feasibility study to test the preliminary effects of Zentangle and Pastel Nagomi on the mental well-being of undergraduate students. The followings were the specific research questions:

1. Feasibility: (a) What are the consent rate, (b) the attrition rate, (c) the reasons for dropout, (d) the intervention compliance rate and (e) the reasons for noncompliance? (f) What suggestions do participants have for the intervention and logistic improvement?
2. Preliminary intervention effect: (a) What are the estimates of the treatment effect of a mixed mode face-to-face and online mode with eight weekly one-hour workshops for undergraduate students on the primary outcomes: improving the psychological state of depression, anxiety and stress; and on the secondary outcomes: improving self-efficacy, emotion, and mindfulness state? (b) What intervention benefits/harms do participants describe in their own words?
3. Qualitative data about feasibility and intervention benefits: (a) What are participants’ explanations for potentially discrepant quantitative results?

## 2. Method

### 2.1. Design

A 3-arm randomized control trial study design was used: one arm with Zentangle workshops, the other arm with Pastel Nagomi Art, while the control group received usual care (i.e., the university provides different mental health workshops and counselling to all students). Focus group interviews followed the interventions to assess the process evaluation and explore the intervention effects.

### 2.2. Participants

A sample size of 10–15 in a group is recommended for a feasibility study (Hertzog, 2008). Chinese-speaking undergraduate students from a university in Hong Kong were invited to participate in the study. Students who had attended or were attending Zentangle or Pastel Nagomi classes were excluded. Recruitment was conducted through emails and promotion posters. Both convenience sampling and snowball sampling methods were used. The application period lasted for 1 week, between 18 January 2021 and 26 January 2021.

### 2.3. Data collection procedure

Ethical approval was obtained from the Institutional Review Board before the commencement of the study. An online questionnaire was sent to the registered students (*N* = 41) with an information sheet and consent form. Details of the university counselling service were provided in the information sheet. After receiving the completed questionnaire, the students who agreed to participate in the study were randomly assigned to three groups: (1) the control group, (2) the Zentangle method, and (3) Pastel Nagomi Art, using computer-generated numbers [0 for the control group; 1 for Zentangle group; and 2 for Pastel Nagomi Art group]. The randomization was conducted by a colleague who was not involved in the study. The participants were then informed of their groupings and intervention schedule. Eight students withdrew at this stage due to timetable clashes, or through failing to respond. Finally, there were 33 participants: 12 in the Zentangle group, 11 in Pastel Nagomi Art, and 10 in the control group. The study took place between 3 February 2021, and 31 March 2021.

### 2.4. Intervention group

This mixed-mode intervention consisted of two face-to-face and six online workshops. A qualified instructor with certified qualifications for teaching both Zentangle and Pastel Nagomi Art was recruited for the workshops. Eight weekly 60-min workshops of Zentangle or Pastel Nagomi Art were held with groups of 3–8 participants. The participants were invited to have face-to-face workshops for the first and last sessions. Social distancing and infection control measures were maintained, and no food and drinks were allowed during the face-to-face workshops. The second to seventh sessions were held in online mode. Zoom, which is a free technology-mediated communication application, was used as the online platform for these meetings.

### 2.5. Control group

The participants in the control group received care as usual at the university. Two weeks after the completion of the study, they were invited to participate in one face-to-face Zentangle or Pastel Nagomi workshop of their own choice.

### 2.6. Outcome measures

To determine the feasibility of the study, (a) consent rate; (b) attrition rate; (c) intervention compliance and acceptance were assessed. To determine the preliminary effect of the intervention, study questionnaire data were collected at the baseline, T0, (before the commencement of the session); T0.5, (immediately after the 4^th^ session); T0.75, (immediately after the 6^th^ session); immediately after the last session, T1; and 1 month after the last session, T2. The questionnaire consisted of eight parts:

1. Demographic information: Students’ personal information, such as age, gender, the form of university entrance, the program of study, year of study, religious affiliations, cumulative GPA, and the number of subjects taken in the current semester, was collected.
2. Perceived psychological distress: the Depression Anxiety Stress Scale (DASS–21) developed by Lovibond and Lovibond (1995) was adopted to measure the participants’ levels of depression, anxiety and stress. This consists of 21 items with three subscales of seven items each (depression, anxiety and stress). Each item was scored on a 4-point Likert scale, ranging from 0 = “Did not apply to me at all” to 3 = “Applied to me very much or most of the time”). The sum of item scores was calculated for each subscale, with a higher score indicating more severe levels of distress. The internal consistency was found acceptable for each subscale and the total scale ranged from (α = 0.80 to 0.93) in an earlier study of undergraduates (Cheung et al., 2020a,b).
3. Self-Efficacy: The participants’ ability to cope with different stressful situations was measured by the 10-item General Self-Efficacy Scale (GSES; Schwarzer and Jerusalem, 1995). A 4-point Likert scale was adopted, ranging from 1 = “Not at all true” to 4 = “Exactly true.” The summation score was used, with a higher score indicating a greater sense of self-efficacy (Schwarzer and Jerusalem, 1995). The internal consistency was found previously to be high, from α = 0.76 to α = 0.90 (Schwarzer and Jerusalem, 1995).
4. Emotion states: The 20-item Positive and Negative Affect Scale (PANAS) was used to measure the participants’ overall emotional states at a particular time (Watson et al., 1988), with 10 items each for Positive Affect (PA) and Negative Affect (NA). Each item was scored on a 5-point Likert Scale ranging from 1 = “Very slightly” or not at all to 5 = “Extremely.” The summation score (10–50) was used for the analysis – for PA, higher scores indicate higher levels of positive affect; for NA, higher scores reported more negative moods. It demonstrated an excellent internal consistency ranging from α = 0.86 to α = 0.90 for PA and from α = 0.84 to α = 0.87 for NA (Watson et al., 1988).
5. Mindfulness state: 12-item revised Cognitive and Affective Mindfulness Scale, revised (CAMS-R) was used to measure participants’ mindfulness states (Feldman et al., 2007). A 4-point Likert scale, ranging from 1 = “Rarely/Not at all” to 4 = “Almost always” was adopted. Items 2, 6, and 7 were reverse-scored. Score summation was used for the analysis, with a higher score indicating higher levels of mindfulness consciousness (Feldman et al., 2007). CAMS-R had shown discriminant validity in a previous study, with a concurrent measure of mindfulness, distress, well-being, emotion regulation and problem-solving approaches (Feldman et al., 2007). Its internal consistency was found acceptable, α = 0.76 (Feldman et al., 2007).
6. Personality: The 10-item Brief Version of the Big Five Personality Inventory (BBF-10) was adopted from Rammstedt and John (2007) to investigate participants’ personalities on five traits, Extraversion, Agreeableness, Conscientiousness, Neuroticism and Openness to Experience. Each subscale consisted of two items with scoring on a 5-point Likert scale, ranging from 1 = “Strongly disagree” to 5 = “Strongly agree.” Items 1, 3, 4, 5, and 7 were reverse-scored. Score summation for each subscale was used to identify the most matched traits for the individuals. The BBF-10 was previously found to have an acceptable internal consistency for each subscale (ranging from α = 0.74–0.89), validity and test–retest reliability with undergraduates (Rammstedt and John, 2007).

Please refer to Table 1 for Cronbach’s alphas of the above-mentioned scales used in this study.

**Table 1:** The Cronbach’s alphas of the scales used in the present study (*N* = 31).

Scale	Baseline	Week 4	Week 6	Week 8	Week 12
	α	α	α	α	α
DASS					
Depression	0.93	0.93	0.93	0.91	0.92
Anxiety	0.78	0.73	0.84	0.78	0.90
Stress	0.88	0.87	0.86	0.85	0.91
General self-efficacy scale	0.86	0.95	0.91	0.95	0.94
Positive Affect	0.93	0.91	0.87	0.90	0.83
Negative Affect	0.94	0.92	0.92	0.91	0.86
Cognitive and affective mindfulness scale revised	0.71	0.78	0.77	0.81	0.77
Brief version of the big five personality inventory					
Extraversion	0.86	0.92	0.86	0.85	0.91
Agreeableness	0.82	0.77	0.90	0.64	0.80
Conscientiousness	0.69	0.63	0.81	0.73	0.80
Neuroticism	0.85	0.89	0.87	0.86	0.89
Openness to experience	0.82	0.78	0.77	0.75	0.74

### 2.7. Focus groups

All participants from the two artwork groups were invited to participate in 45–60 min focus group interviews one month after the workshop sessions, to share their acceptance of and satisfaction with their experiences.

## 3. Data analysis

### 3.1. Quantitative data analysis

The Statistical Software Package for the Social Sciences (SPSS), version 26.0 was used for data analysis. Descriptive statistics, namely frequencies, means, and standard deviations, were used to analyze the study variables. The Kruskal-Wallis and Wilcoxon signed-ranks tests were used to analyze the time-point differences between groups and within groups, respectively. The significance level was set at *p* < 0.05. The statistical power to warrant hypothesis testing was not required due to the small sample sizes for the feasibility, pilot, and exploratory studies (Lee et al., 2014).

### 3.2. Qualitative data analysis

The focus group interviews were audio-recorded. The audio recordings were transcribed verbatim in Chinese and then imported into NVivo Pro 12 for data management and analyzed by two coders (EM, CH) using theoretical thematic analysis procedures (Braun and Clarke, 2006). The themes were identified by deriving from “the explicit meaning of the data … [with]the analyst not looking for anything beyond what a participant said” (Braun and Clarke, 2006, p. 84). The coding was guided by research questions related to intervention benefits, harm as well as explaining and supplementing quantitative results. The two coders coded the transcripts separately and discussed the coding to ensure agreement on a basic set of codes and categories.

## 4. Results

### 4.1. Participants

Table 2 summarizes the participants’ demographic and characteristics at baseline. 90.9% (*n* = 30) were females and 84.8% (*n* = 28) were transfer students, who were community college graduates admitted to the undergraduate program (Cheung et al., 2020a,b). 51.5% (*n* = 17) were Year 1 students, 30% (*n* = 10) of them were studying in Year 2, 6.06% (*n* = 2) were Year 3 students and 8.25% (*n* = 4) were Year 4 students. Significant differences for BBF10-neuroticism (*p* < 0.05) and BBF10-openness (*p* < 0.05) were found between the participants in the Pastel Nagomi Art and control groups at baseline. Otherwise, there were no significant differences in demographic or other variables found among the group.

**Table 2:** The characteristics of study participants at baseline (*N* = 33).

Students	Pastel Nagomi Art group		Zentangle group		Control group		Chi-square
	n = 11		n = 12		n = 10		
	*n*	%	*n*	%	*n*	%	*p*-value
Gender							
Male	1	9.1	0	0.0	2	20	
Female	10	90.9	12	100.0	8	80	
Age							0.35
19 to 20	5	45.5	4	33.3	1	10	
21 to 22	3	27.3	6	50.00	3	30	
23 to 24	2	18.2	2	16.7	4	40	
≧25	1	9.1	0	0.00	2	20	
Form of university entrance							1.000
Non-Transfer	2	18.2	2	16.7	1	10	
Transfer	9	81.8	10	83.3	9	90	
Religious beliefs							1.000
None	9	81.8	10	83.3	9	90	
Christian	2	18.2	2	16.7	1	10	
Year of study							0.73
Year 1	6	54.5	7	58.3	4	40	
Year 2	3	27.3	4	33.3	3	30	
Year 3	0	0.0	0	0.00	2	20	
Year 4	2	18.2	1	8.3	1	10	
cGPA							0.111
Mean	3.2		2.9		3.2		
SD	0.3		0.5		0.3		
Number of subjects							0.39
Mean	5.6		6.0		5.1		
S.D	0.9		1.3		1.9		
Min.	4		4		0		
Max.	7		8		6		
Personality	Mean	S.D	Mean	S.D	Mean	S.D	Kruskal-Wallis
Extraversion	5.7	0.9	5.3	1.4	5.7	1.2	0.61
Agreeableness	6.7	0.9	7.1	1.6	7.2	0.8	0.56
Conscientiousness	6.0	1.7	5.3	1.0	5.1	1.5	0.48
Neuroticism	7.0^#^	1.1	7.0	1.9	6.0^#^	0.7	0.092
Openness to experience	6.9^#^	1.6	6.0	1.5	5.4^#^	1.1	0.090

# Significant differences on BBF10-neuroticism (*p* < 0.05) and BBF10-openness (*p* < 0.05) between Pastel Nagomi Art and control groups.

### 4.2. Study feasibility

**Figure 3:**
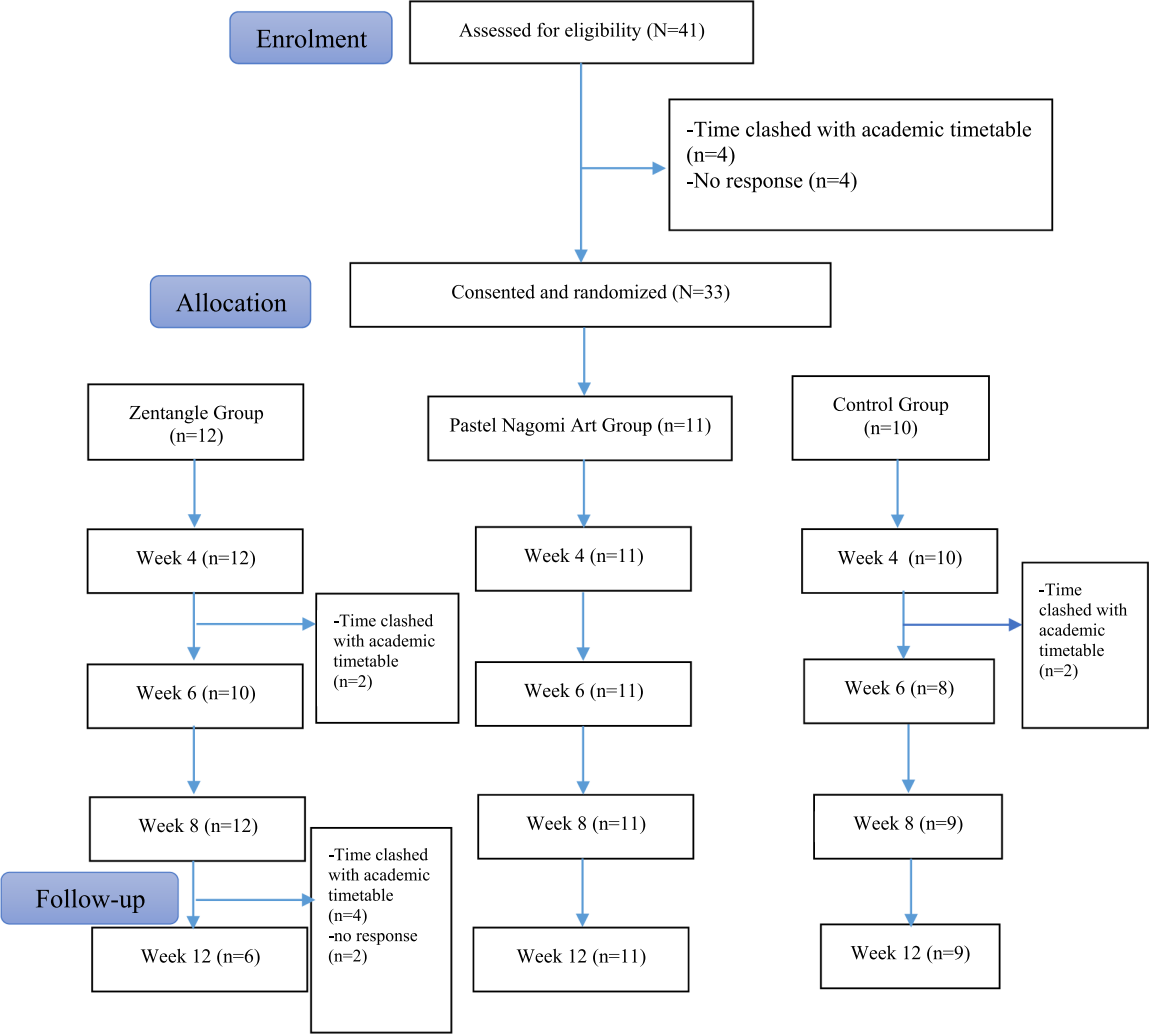
Consort diagram of the study.

A total of 41 students registered for the study, 33 agreed to participate, and 31 completed the study. Thus, the consent rate was 80.5% while the attrition rate was 6.06%. Measurement completion rates at week 8 were 100% for the three groups. The attrition occurred only in the Zentangle group. Figure 3 shows the study consort diagram.

In the Pastel Nagomi Art group arm, 100% (*n* = 12) completed the 8-week program. Of the 12 participants in the Zentangle group arm, 83.3% (*n* = 10) attended the week-6 workshop, and 100% attended the other 7 workshops.

Twenty participants attended the focus groups, 11 from the Pastel Nagomi Art group and 9 from the Zentangle group. In the focus group, participants expressed great satisfaction with both art forms. All expressed having no strong preference for the modes of the workshops. 72.7% (*n* = 8) of the Pastel Nagomi Art group participants and 77.8% (*n* = 7) of the Zentangle group did say that the face-to-face mode provided better interaction with the instructor and classmates to build social networks and the feeling of being supported, and hence being able to receive clear instruction about the steps of the artwork. On the other hand, 27.3% (*n* = 3) of Pastel Nagomi Art group participants and 22.2% (*n* = 2) Zentangle group participants indicated that the online mode allowed them to use the art materials and tools more conveniently and in a more relaxed way at home. Most participants in the Pastel Nagomi Art group suggested that the workshop length could be extended to 75 min. The majority of participants (*n* = 16, 80%) agreed the workshop should start at the beginning of the semester but some of them (*n* = 4, 20%) suggested they could be held after the examination period.

### 4.3. Preliminary intervention effects

#### 4.3.1. Quantitative results

In general, Tables 3, 4 demonstrate that, compared with the control group, both intervention groups show promising improvement trends within the eight-week intervention period and this was sustained for another 4 weeks after the intervention but for different outcome variables.

**Table 3:** Intervention effect on perceived psychological distress for within-groups compared with baseline and between-groups compared with the control group.

		Pastel Nagomi Art group				Zentangle group				Control group				Between-group comparison	
		Mean	S.D	Within group change^@^	Within group	Mean	S.D.	Within group change^@^	Within group	Mean	S.D	Within group change^@^	Within group	Pastel Nagomi Art group vs. control group	Zentangle group vs. control group
					*p*				*p*				*p*	*p*	*p*
Depression															
	Baseline	10.8	12.7			12.8	8.9			9.3	6.2			0.82	0.43
	Week 4	11.8	11.1	1.0	0.73	11.0	7.5	−1.8	0.21	10.4	9.7	1.1	0.95	0.62	0.50
	Week 6	10.5	8.9	−0.3	0.91	11.4	11.3	−1.4	0.191	9.5	8.8	0.2	0.56	0.82	0.85
	Week 8	10.0	9.5	−0.8	0.76	9.7	8.1	−3.2	0.034*	8.4	9.5	−0.9	0.078	0.160	0.44
Week-12 Follow-up		8.9	8.0	−2.9	0.49	8.0	8.1	−4.8	0.31	13.0	12.5	3.7	0.88	0.62	0.47
Anxiety															
	Baseline	9.3	7.4			8.3	6.8			10.0	4.9			0.70	0.50
	Week 4	9.1	7.0	−0.2	0.87	9.7	5.6	1.4	0.45	9.8	5.7	−0.2	0.67	0.70	0.37
	Week 6	10.0	7.4	0.7	0.98	10.2	9.6	1.9	0.98	8.5	7.2	−1.5	0.20	0.32	0.56
	Week 8	7.5	6.5	−1.8	0.063	8.8	6.6	0.5	0.94	6.7	5.3	−3.3	0.031*	0.130	0.150
Week-12 Follow-up		8.2	6.2	−1.1	0.56	5.3	5.0	−3.0	0.66	15.3	11.7	5.3	0.44	0.30	0.31
Stress															
	Baseline	12.7	10.8			11.0	6.3			12.4	6.4			0.91	0.57
	Week 4	12.5	9.3	−0.2	0.84	14.0	7.5	3.0	0.21	11.6	6.1	−0.8	0.55	0.70	0.153
	Week 6	12.7	8.9	0.0	0.78	15.0	9.6	4.0	0.48	13.5	9.1	1.1	0.88	0.86	0.52
	Week 8	12.5	10.4	−0.2	0.85	13.5	6.2	2.5	0.21	11.8	6.3	−0.7	0.23	0.43	0.075
Week-12 Follow-up		10.2	7.0	−2.5	0.31	14.7	8.9	3.7	0.63	17.5	10.5	5.1	0.63	0.27	1.000

* *p* < 0.05.

^#^Kruskal-Wallis and Wilcoxon signed-ranks tests were used for between-group and within-group analysis, respectively.

@ For the within-group change, the comparison is between that moment and the baseline.

**Table 4:** Intervention effect on self-efficacy, emotional states and mindfulness state for within-groups compared with baseline and between-groups compared with the control group.

		Pastel Nagomi Art group				Zentangle group				Control group				Between-group comparison	
		Mean	S.D	Within group change^@^	Within group	Mean	S.D.	Within group change^@^	Within group	Mean	S.D	Within group change^@^	Within group	Pastel Nagomi Art group vs. control group	Zentangle group vs. control group
					*p*				*p*				*p*		*p*
Self-efficacy															
	Baseline	27.1	4.8			25.7	4.3			26.7	2.6			0.616	0.54
	Week 4	28.8	5.8	1.7	0.24	25.4	5.7	−0.3	1.000	24.8	5.5	−1.9	0.53	0.182	0.57
	Week 6	27.4	4.0	0.3	0.44	26.5	5.6	0.8	0.59	29.0	3.2	2.3	0.13	0.251	0.35
	Week 8	26.7	5.5	−0.4	0.61	24.3	6.5	−1.4	0.27	27.6	4.7	0.9	0.34	0.222	0.134
Week-12 Follow-up		29.1	5.6	2.0	0.190	27.0	3.1	1.3	1.000	25.9	6.8	−0.8	0.75	0.439	0.89
Positive Affect															
	Baseline	28.8	7.4			25.8	6.6			31.4	7.0			0.402	0.102
	Week 4	27.6	7.9	−1.2	0.47	28.8	5.8	3.0	0.25	24.8	6.2	−6.6	0.008*	0.137	0.002*
	Week 6	28.0	6.1	−0.8	0.63	25.0	5.9	−0.8	0.23	24.5	7.0	−6.9	0.016*	0.037*	0.22
	Week 8	28.5	7.5	−0.4	0.79	25.7	6.1	−0.2	0.44	26.2	6.0	−5.2	0.023*	0.050	0.103
Week-12 Follow-up		30.1	7.6	1.3	0.45	27.8	4.9	2.0	1.000	29.8	4.9	−1.7	0.56	0.173	0.56
Negative Affect															
	Baseline	27.1	7.7			22.7	8.0			26.0	9.0			0.703	0.22
	Week 4	25.7	9.0	−1.4	0.28	26.4	7.4	3.7	0.14	20.2	5.4	−5.8	0.031	0.168	0.006*
	Week 6	24.9	9.4	−2.2	0.047*	24.5	7.6	1.8	0.95	20.0	6.4	−6.0	0.094	0.361	0.12
	Week 8	24.5	7.2	−2.6	0.074	25.7	8.2	3.0	0.69	20.7	3.9	−5.3	0.063	0.803	0.069
Week-12 Follow-up		21.6	4.9	−5.5	0.006*	27.3	9.2	4.7	0.88	25.3	5.6	−0.8	0.31	0.234	0.52
Mindfulness state															
	Baseline	30.9	4.3			30.6	4.6			30.7	3.4			0.88	0.80
	Week 4	30.2	4.9	0.73	0.23	29.4	4.6	1.17	0.34	29.1	3.8	1.78	0.48	0.82	0.97
	Week 6	30.5	4.8	0.36	1.000	28.5	5.6	2.00	0.25	30.6	2.3	−0.29	1.000	0.89	0.33
	Week 8	31.3	5.3	−0.36	0.87	28.7	5.3	1.92	0.125	29.6	4.4	0.88	1.000	0.84	0.31
Week-12 Follow-up		31.5	4.9	−0.55	0.98	31.0	3.8	2.17	0.063	29.1	5.3	0.86	0.69	0.75	0.31

* *p* < 0.05.

^#^Kruskal-Wallis and Wilcoxon signed-ranks tests were used for between-group and within-group analysis, respectively.

@ For the within-group change, the comparison is between that moment and the baseline.

##### 4.3.1.1. Within-group results

The Pastel Nagomi art group had a significant decrease in negative affect at week 6 (mean change = −2.2; *p* = 0.047) and at week-12 follow-up (mean change = −5.5; *p* = 0.006; Table 4) as compared with baseline. Moreover, the Zentangle group showed a significant decrease in depression at week 8 (mean change = −3.2; *p* = 0.034; Table 3). For the control group, there was a significant reduction in positive affect on weeks 4 (mean change = −6.6; *p* < 0.001), 6 (mean change = −6.9; *p* = 0.016) and 8 (mean change = −5.2; *p* = 0.023; Table 4). Although improvement was shown in anxiety at week 8 (mean change = −3.3; *p* = 0.003; Table 3), this reduction not only did not sustain at the week-12 follow-up, but also was worse than the baseline (Table 3).

##### 4.3.1.2. Between-group results: Pastel Nagomi art group vs. control group

Pastel Nagomi art group presented a significantly better result in retaining positive affect (mean change = −0.80) than the control group (mean change = −6.9) at week 6 (*p* = 0.037; Table 4). This retention could be further observed at week 12 (mean change = +1.3).

##### 4.3.1.3. Between-group results: Zentangle group vs. control group

Similar to the Pastel Nagomi art group, the Zentangle group (mean change = +3.0) also showed a significant increase in positive affect than the control group (mean change = −6.6) at week 4 (*p* = 0.002), with better retention at week 12 (Table 4). On the other hand, the control group (mean change = −5.8) had a significantly greater decrease in negative affect than the Zentangle group (mean change = +3.7) at week 4 (*p* = 0.006; Table 4).

In summary, the within-group analyses showed that the Pastel Nagomi art group had significantly decreased negative affect at weeks 6 and week 12. Moreover, the Zentangle group showed a significant decrease in depression at week 8. From the between-group analyses, compared with the control group, the Pastel Nagomi art group had a significant improvement in retaining positive affect at week 6. This retention could be further observed at week 12. Similar to the Pastel Nagomi art group, the Zentangle group had a significant increase in positive affect at week 4, with better retention at week 12.

For the control group, on the other hand, the within-group analyses showed that there was a significant reduction in positive affect on weeks 4, 6 and 8. Although improvement was shown in anxiety at week 8, this reduction not only did not sustain at the week-12 follow-up, but also was worse than the baseline. The between-group analyses found that the control group had a greater decrease in negative affect at week 4 than the Zentangle group.

#### 4.3.2. Qualitative results

Twenty participants attended the focus groups, 11 from the Pastel Nagomi Art group and 9 from the Zentangle group. Five focus groups were conducted with 2 to 6 participants for each group. Two coders achieved 88.7% agreement in the first draft. After that, the coders discussed the diversity and coded the themes again. The codes achieved 100% agreement at the second coding. The percentage agreement by adding up the identical codes that the coders applied and dividing the results by the total number of codes which can reflect the reliability of the protocol as the protocol involves a simple coding task (Feng, 2014). No participants indicated any harmful effects. The coding for intervention benefits resulted in the identification of three themes and seven sub-themes (Table 5). For the first “*enjoyment of artwork process*,” the participants from both intervention groups described that they felt a *relaxed and joyful* atmosphere during the artwork process. This suggests that the workshop provided quality time for them to relax and manage their stress. Some participants in the Pastel Nagomi Art group expressed that they felt relieved from daily stress when they used the skills of drawing Pastel Nagomi Art. Some participants from the Zentangle Art group said that they felt the time went fast during the workshop. Likewise, a few participants from the Pastel Nagomi Art group felt that the face-to-face intervention session provided them with an opportunity to *build a social network* and hence relieve stress. Moreover, some participants from both groups reflected they felt the intervention workshops enhanced their attention, through concentrating on the drawing and ignoring the stressors during the intervention workshops.

**Table 5:** Qualitative Findings: Intervention Benefits.

		Pastel Nagomi Art group	Zentangle group
Theme	Sub-themes	Examples	Examples
Enjoyment of the artwork process	Relaxation and joyfulness	I felt happy when I saw my artwork. This feeling made me feel relaxed. I also felt happy with my improvement in the artwork.	I felt relaxed! Even though I spent an hour drawing, but I did not notice it as the time passed so fast. The relaxation during the time of the drawing was invaluable and I was happy.
	Focus	Focusing on repetitive scraping and rubbing with my fingers made me feel relaxed. Focusing on the artwork process made me feel good.	I could solely focus on Zentangle when I was drawing. Probably, I was so focused on drawing that I neglected other businesses.

	Social networking	I also enjoyed the moment of drawing with others. I believe that it is the social bonding which helps relieving my stress.	I felt more relaxed during the face-to-face sessions (the first and last session) which I can interact with the instructor and others.
Proud of the artwork	Satisfaction	I felt a sense of satisfaction after finishing the piece.	I found my work was really beautiful when it was viewed from a distance. The achievement I felt was actually greater after completing the piece.
	Willing to share	I would share my artwork with friends through WhatsApp, and I got their compliments. Then, I shared them with my family hoping that they would admire my work.	I sent my finished artwork to my friends, they praised it. They even asked whether I purchased it from somewhere else. I felt that I was so great.
Personal growth	A renewed sense of self	We had to learn more about ourselves before getting changed. Drawing is a good way to get to know myself better. I believe that drawing is beneficial to personal growth.	It could help me to be aware of my stress.
	A new relaxation method	I considered drawing as an alternative way to relax. I used to relax through drawing and now I have acquired a new drawing technique to relax.	In my spare time after drawing class, I took out a piece of paper and draw some patterns that I have learnt in the class. I found a new way to relax.

For the second theme “*pride in the artwork*,” the participants from both intervention groups expressed that they *felt satisfied and confident* after the drawing. A few participants from both the Pastel Nagomi Art and Zentangle Art groups revealed that they *felt fulfilled* when seeing their art. Furthermore, the majority revealed that they were *willing to share* their artwork products with their friends and families. Both groups shared that they felt satisfied when their friends and families praised their artwork.

Through the intervention workshops, the participants felt they had experienced *personal growth*, as the third theme. A few participants from each intervention group reflected that the workshops allowed them to *renew their sense of self* through reflecting on themselves and releasing their emotions. Moreover, some participants from each group indicated that they had *learned a new relaxation skill* during the intervention to assist them to manage their stress.

## 5. Discussion

### 5.1. The feasibility of the study

To the best of our knowledge, this is the first study to investigate the feasibility and preliminary effects of a mixed-mode (face-to-face and online) intervention of Pastel Nagomi Art and Zentangle Art on improving the mental health of undergraduate students during the COVID-19 pandemic. The present study achieved an acceptable consent rate, demonstrating acceptable recruitment. In addition, high attendance rates in both intervention groups suggested excellent acceptability of the intervention. Timetable clashes were the main reasons for absence from workshops. Furthermore, the focus-group interview participants provided supporting evidence of their acceptance of both artwork interventions and the modes of the intervention. Compared with previous studies with the consent rate and attendance rate ranged from 56.3–70.2% (Coholic et al., 2018) and 50.3–95% (Lee, 2021) respectively, the present study has demonstrated its high feasibility to pursue a full-scale study.

In the current study, a mixed mode (face-to-face and online) was used which is probably the first of its kind. Most of the artwork interventions in previous studies were conducted merely face-to-face (Kim, 2013; Chen et al., 2016; Karpavičiūtė and Macijauskienė, 2016; Campenni and Hartman, 2020; Newland and Bettencourt, 2020; Hsu et al., 2021) or online modes (Sit et al., 2022). In terms of dose, frequency and duration, this present study is comparable with previous work. According to a recent systematic review of the effectiveness of mindfulness-based artwork therapy for managing symptoms of anxiety, depression, and fatigue, among 14 studies, the doses (i.e., lengths of sessions) ranged from 45 to 180 min, the frequencies (i.e., the time intervals between sessions) were weekly or bi-weekly, and the durations ranged from two to 13 weeks (Newland and Bettencourt, 2020). In this review, 11 studies (78.57%) conducted interventions weekly for eight weeks (Newland and Bettencourt, 2020), which the present study also adopted. There were some variations. Hsu et al. (2021) conducted a face-to-face Zentangle art study for healthcare workers with one four-hour session; while Sit et al. (2022) conducted Zentangle art in online mode with weekly 30-to 90-min sessions for 11 weeks. The doses of the interventions might have varied because of the types of artwork, for instance, 90 min for creative art-making (Burns and Waite, 2019) and 180 min for content-picture drawing (Monti et al., 2013). The doses of Zentangle art ranged from 60 (Chen et al., 2016) to 90 min (Chia et al., 2020). It is worth noting that in the interviews the participants suggested extending each session to 75 min. Future studies should explore the dose, frequency and duration of interventions further.

### 5.2. The effectiveness of artwork on mental well-being

The results of this study support positive intervention effects, as both intervention groups improved in regard to depression, and positive affect, compared with the control group. Furthermore, as compared with the control group, the Pastel Nagomi art had a more sustainable effect on stress as noted in week 12. Karpavičiūtė and Macijauskienė, 2016 conducted an RCT and found that silk painting artwork could improve mental well-being, reducing stress and fatigue and increasing a sense of community at work among nurses and nursing assistants. Furthermore, previous studies also found art making with paint, markers, and collage materials on canvas material reduced stress in female counsellors (Ifrach and Miller, 2016). Thyme et al. (2007) indicated that painting and drawing helped to reduce depression symptoms in depressed women and relieved depression, perceived stress and parenting stress in Korean mothers of children with disabilities (Lee, 2021). Coloring mandalas can also help to reduce anxiety and negative affect and increase positive affect in undergraduates (Campenni and Hartman, 2020). Moreover, Zentangle art can improve self-esteem and reduce social interaction anxiety in patients with psychosis (Chen et al., 2016) and enhance psychological and family well-being (Sit et al., 2022). One previous study supported the present study’s finding, reporting that a four-week creative art program, including water-coloring, collage making, beading and knitting, was effective in enhancing the mental well-being among women (Fraser and Keating, 2014).

Although improvement trends were observed, there were no significant improvements in stress, anxiety, mindfulness and self-efficacy. One reason was the small sample size in this study. The improvement trends suggested that it might take time for the effects of both artworks on mental well-being through enjoyment of the artwork process, pride in the artwork and personal growth to develop. The qualitative exploration of this study supported this suggestion. The participants in both intervention groups experienced a relaxing and joyful atmosphere, being focused and establishing social networks. They pointed out that the eight artwork sessions provided them with regular weekly one-hour sessions away from stressors and allowed them to take a break and refresh their minds during the intervention. Regular practice of relaxation techniques can help individuals to manage their stress at moderate emotional levels (Pfeiffer, 2001). Moore (2013) reported that a repetitive act of drawing patterns or lines, similar to the Zentangle art, helped individuals get into a mindfulness state and generate self-healing processes to reduce stress. Karpavičiūtė and Macijauskienė, (2016) found that finger painting which was similar to the concept of Pastel Nagomi art could improve the state of mindfulness. Tactile sensation, mindfulness state and the experience of being present are related to cognitive processes (Grossman, 2011; Hinz, 2020). Developing a state of mindfulness could be effective in decreasing mood disturbance and stress (Brown and Ryan, 2003). In addition, Salzano et al. (2013) reported that group painting activities helped hospice caregivers to increase social support and hence reduce their stress, which is consistent with our findings.

Moreover, it was important that the participants were keen to share their artwork products with others or on social media, which serves as evidence they felt satisfied with their products. The participants expressed their enjoyment of receiving compliments from others on their artwork. Lin Brostrom et al. (2020) found that sharing the artwork could provide a positive new topic and an opportunity for the participants to interact with others via technologies such as text messages and social media. Choi and Choung (2021) also found that women who used social media for social connections had increased satisfaction with life during the COVID-19 pandemic. The present study supported previous findings that artworks can improve subjective well-being by providing positive self-images and hope, enhancing satisfaction with daily life, and expanding contact with the outside world (Reynolds et al., 2008). The satisfactory artwork products and sharing behaviours could further enhance self-esteem and self-efficacy.

Furthermore, the participants in both intervention groups encountered personal growth with a renewed sense of self and learned a new relaxation skill. They reflected that they had increased their awareness of their emotions, especially the changes that occurred to their negative affect, which added support to the finding that the Zentangle art group participants’ negative affect was higher than that of the control group participants at week 4. The Zentangle art group participants had increased their awareness of their emotions after the intervention, but they may still have tried to deal with the negative affect. Emotional awareness was found as an essential component of effective emotional regulation (Gratz and Roemer, 2004). The repetitive movement in both artworks could help the participants get into a state of mindfulness to increase their awareness of the present (Moore, 2013; Karpavičiūtė and Macijauskienė, 2016). This increased awareness helps individuals reconsider threatening situations and regulate emotional responses (Modinos et al., 2010; Prazak et al., 2012). Learning a new relaxation skill allowed the participants to acquire a new method to release stress for long life, not only for the short period of time of the study.

### 5.3. Limitation

This study had some limitations. An imbalance number of online vs. face-to-face sessions might affect the study outcomes. This study was conducted during the COVID-19 pandemic, social distancing was reinforced. Thus, to minimize face-to-face contact, only two face-to-face workshops were conducted. Although the participants did not have a strong preference for the modes of the workshops, about three-fourths of them preferred face-to-face. Further studies can consider four online and four face-to-face workshops in the post-pandemic situation. The two-time points of data collection at weeks 4 and 6 during the 8-week intervention could be another limitation. As mentioned, a recent systematic review found that the duration of interventions ranged from two to 13 weeks (Newland and Bettencourt, 2020). With this reference, the intention of collecting data at these two-time points was to test the effect of the interventions in a short duration. However, repeated measures might induce survey fatigue. In addition, according to the qualitative results of this study, participants’ confidence has been improved. In this study, the Rosenberg (1965) 10-item self-esteem scale was used but low Cronbach’s alpha values at different time points were registered (0.01 < α < 0.66). These low values could be due to the small sample size. Due to the low values, the self-esteem scale was not included in the analysis. Future studies should re-assess the psychometric properties of this scale for this university student population, and other scales can also be used to verify the impact of these two studied artworks on self-esteem. This study involved students from one university which affects the generalizability of the study results.

## 6. Conclusion

The quantitative and qualitative findings of the present study have supported that Zentangle and Pastel Nagomi Art are effective in improving mental well-being. Both artworks might also build essential stepping stones for enhancing mindfulness, self-efficacy and self-esteem. Furthermore, the feasibility of this study also demonstrated acceptable recruitment, consent, attendance and retention rates. For future large-scale studies, the duration of each session can be extended to 75 min to allow the participants more time to enjoy the artwork. Furthermore, longer intervention periods or additional interventions may be needed to sustain the positive intervention effect.

## Data availability statement

The original contributions presented in the study are included in the article/supplementary material, further inquiries can be directed to the corresponding author.

## Ethics statement

The study involving human participants were reviewed and approved by the Institutional Review Board of the Hong Kong Polytechnic University. The patients/participants provided their written informed consent to participate in this study.

## Author contributions

KC planned the study, coordinated the study, and revised the manuscript. KYM taught the art workshops, drafted and revised the manuscript. HT conducted data analysis. NHL and KYL coordinated the art workshops. SWH coded the qualitative result. All authors contributed to the article and approved the submitted version.

## Funding

The study was funded by the University Grant Committee (UGC) Funding Scheme for Teaching and Learning Related Proposals (2016-19 Triennium; PolyU6/T&L/16–19).

## Conflict of interest

The authors declare that the research was conducted in the absence of any commercial or financial relationships that could be construed as a potential conflict of interest. All authors contributed to the article and approved the submitted version.

## Publisher’s note

All claims expressed in this article are solely those of the authors and do not necessarily represent those of their affiliated organizations, or those of the publisher, the editors and the reviewers. Any product that may be evaluated in this article, or claim that may be made by its manufacturer, is not guaranteed or endorsed by the publisher.
